# Surface Engineering of Anodic WO_3_ Layers by In Situ Doping for Light-Assisted Water Splitting

**DOI:** 10.1021/acsami.4c02927

**Published:** 2024-07-05

**Authors:** Karolina Syrek, Sebastian Kotarba, Marta Zych, Marcin Pisarek, Tomasz Uchacz, Kamila Sobańska, Łukasz Pięta, Grzegorz Dariusz Sulka

**Affiliations:** † Faculty of Chemistry, Jagiellonian University, Gronostajowa 2, 30-387 Krakow, Poland; ‡ Laboratory of Surface Analysis, Institute of Physical Chemistry, Polish Academy of Sciences, Kasprzaka 44/52, 01-224 Warsaw, Poland

**Keywords:** tungsten oxide, anodization, in situ doping, nanostructured morphology, OER, photoelectrochemical properties

## Abstract

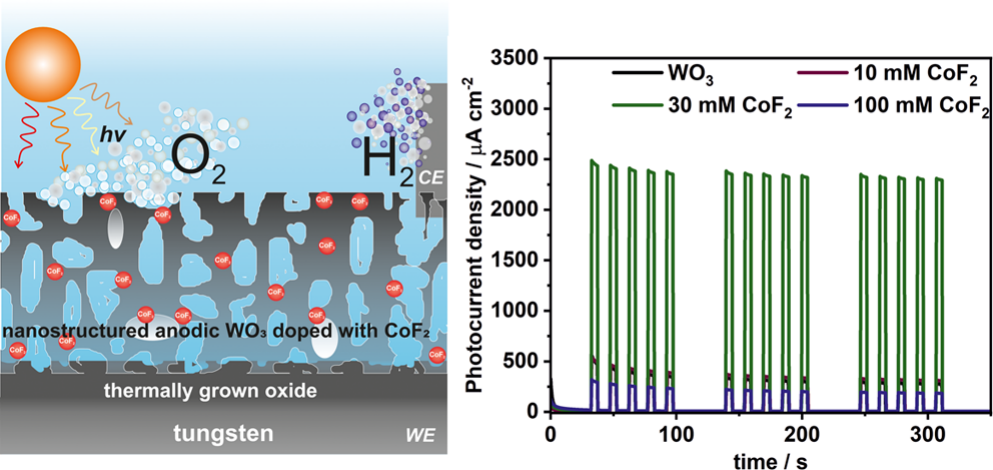

This study presents a novel approach to fabricating anodic Co–F–WO_3_ layers via a single-step electrochemical synthesis, utilizing cobalt fluoride as a dopant source in the electrolyte. The proposed in situ doping technique capitalizes on the high electronegativity of fluorine, ensuring the stability of CoF_2_ throughout the synthesis process. The nanoporous layer formation, resulting from anodic oxide dissolution in the presence of fluoride ions, is expected to facilitate the effective incorporation of cobalt compounds into the film. The research explores the impact of dopant concentration in the electrolyte, conducting a comprehensive characterization of the resulting materials, including morphology, composition, optical, electrochemical, and photoelectrochemical properties. The successful doping of WO_3_ was confirmed by energy dispersive spectroscopy (EDS), X-ray diffraction (XRD), Raman spectroscopy, photoluminescence measurements, X-ray photoelectron spectroscopy (XPS), and Mott–Schottky analysis. Optical studies reveal lower absorption in Co-doped materials, with a slight shift in band gap energies. Photoelectrochemical (PEC) analysis demonstrates improved PEC activity for Co-doped layers, with the observed shift in photocurrent onset potential attributed to both cobalt and fluoride ions catalytic effects. The study includes an in-depth discussion of the observed phenomena and their implications for applications in solar water splitting, emphasizing the potential of the anodic Co–F–WO_3_ layers as efficient photoelectrodes. In addition, the research presents a comprehensive exploration of the electrochemical synthesis and characterization of anodic Co–F–WO_3_, emphasizing their photocatalytic properties for the oxygen evolution reaction (OER). It was found that Co-doped WO_3_ materials exhibited higher PEC activity, with a maximum 5-fold enhancement compared to pristine materials. Furthermore, the studies demonstrated that these photoanodes can be effectively reused for PEC water-splitting experiments.

## 1 Introduction

Photoelectrochemical (PEC) water splitting under solar radiation is considered a promising strategy for producing hydrogen gas, which can serve as a highly energy-dense fuel with clean combustion byproducts.^1^ These systems rely on efficient and stable photoelectrodes, with the predominant focus in the field aimed at addressing low efficiency, limited long-term stability, and applicability restricted to the ultraviolet radiation region.^2−7^ Anodic metal oxides show promise for this purpose due to their unique geometry,^8,9^ relatively high surface area,^10^ and properties such as light absorption ability and generation of charge carriers under illumination.^11^ Furthermore, photoelectrodes based on anodic oxides do not require elaborate preparation because of the relatively good adhesion between the porous film and a conductive metal, as well as its perpendicular orientation to the substrate, which facilitates an efficient electron transfer path.^12^ Among the many anodic oxide films potentially applicable in PEC cells for water decomposition, tungsten oxide deserves special attention due to its narrower band gap of 2.8 eV^13^ (in comparison to commonly used TiO_2_ nanotubes with ∼3.2 eV).^14^ However, its absorption abilities are still predominantly confined to the UV light region. Improvement of PEC properties of anodic oxide layers can be achieved through doping^15^ and surface functionalization^16^ (such as depositing cocatalyst,^17^ other semiconductors,^18,19^ polymers,^20^ or dyes^6^). In most cases, these modifications are implemented as post-treatment procedures (e.g., electrochemical deposition^19^) followed by annealing^18^ under precisely chosen conditions. Generally, doping semiconducting material with metals (e.g., Fe, Cu, Pd, Co)^21^ and/or nonmetals (e.g., C, N, F)^22^ can lead to the formation of an intermediate energy band^15^ or narrow the band gap of the desired material,^23^ influencing the overall PEC performance.

Doped anodic films are primarily produced by the anodization of metal alloys,^24−26^ annealing in suitable conditions (e.g., in the presence of NH_4_OH,^27^ ammonia^28^), or by employing the so-called in situ anodization,^15^ where a suitable precursor is introduced into the electrolyte.^29^ Approaches reported in the literature are predominantly associated with the modification of TiO_2_ nanotubes through in situ anodization of Ti. These methods involve the use of metal salts (e.g., CuSO_4_, AgNO_3_)^30^ or other precursors with relatively large anions, such as ferricyanide,^31^ WO_4_^2–^, Al(OH)_4_^–^, SiO_3_^2–^, MoO_4_^2–^. However, it is essential to note that the presence of large anions in precursors may significantly limit the number of embedded ions within the anodic layer.^32^

Therefore, we propose the in situ doping of anodic tungsten oxide using cobalt fluoride as a dopant source in the electrolyte. Considering the high electronegativity of fluorine, it is anticipated that CoF_2_ will remain stable throughout the entire process. Moreover, the mechanism of formation of the nanoporous layer (resulting from the dissolution of the anodic oxide in the presence of F^–^)^13^ is expected to facilitate the effective incorporation of these species into the film. This paper marks the pioneering introduction of a single-step electrochemical synthesis for Co–F–WO_3_ anodic layers endowed with catalytic properties toward the photoelectrochemical oxygen evolution reaction (OER). The study delves into the influence of dopant concentration in the electrolyte and the resulting materials undergo a comprehensive characterization encompassing their morphology, composition, optical, electrochemical, and photoelectrochemical properties.

## 2 Experimental Section

### 2.1 Materials Synthesis

Anodic WO_3_ layers were synthesized by single-step anodic oxidation of metallic tungsten (99.95%, 0.2 mm thick, GoodFellow) in an electrolyte containing 1 M (NH_4_)_2_SO_4_ and 0.075 M NH_4_F at the constant stirring speed of 250 rpm and at the temperature of 25 °C. A constant potential of 50 V was applied for 4 h.^13,33^ In situ doping with Co was carried out by introducing 10, 30, or 100 mM CoF_2_ to the electrolyte. For sample preparation, a double-wall two-electrode cell was used, in which a Pt mesh and W foil served as a cathode and anode, respectively. During each anodization, the current flowing in the cell was recorded using a Picotest M-3500A multimeter. Subsequently, anodic films were annealed in air at 500 °C for 2 h with a heating rate of 2 °C·min^–1^,^34^ using a muffle furnace (FCF 5SHM Z, Czylok).

### 2.2 Materials Characterization

Nanostructured Co-WO_3_ layers were characterized using a field emission scanning electron microscope (FE-SEM/EDS, Hitachi S-4700 with a Noran System 7). The analysis of morphological features was conducted based on SEM images, which were further processed using CorelDraw software. The distribution graph was constructed using the Kernel density estimation^35^ and presented alongside wall size values for normalized intervals as the rug plot on horizontal axis.

X-ray diffraction (XRD) patterns were obtained using a Rigaku Mini Flex II diffractometer, employing monochromatic Cu Kα radiation (λ = 1.5418 Å). The measurements covered a 2θ range from 10 to 80°, with a step size of 5° min^–1^.

The Auger electron spectrometer/X-ray photoelectron spectrometer (AES/XPS) (Microlab 350, Thermo Electron) was used for monitoring the chemical composition, utilizing the XPS functions of the device with a lateral resolution of 2 × 5 mm^2^. XPS spectra were excited using Al Kα (*h*ν = 1486.6 eV) radiation as a source. Survey and high-resolution spectra were recorded with 100 and 40 eV pass energy, respectively. A smart background subtraction was applied to obtain the XPS signal intensity. The peaks were fitted using an asymmetric Gaussian/Lorentzian mixed function. The measured binding energies were corrected with reference to the energy of C 1s at 284.7 eV. Data acquisition and processing were carried out using Avantage-based data system software (Version 5.9911, Thermo Fisher Scientific). UV–vis diffuse reflectance spectra (DRS) were acquired using a Lambda 750S spectrophotometer (PerkinElmer) equipped with an integrating sphere. Measurements were conducted within the wavelength range of 250–820 nm, with a step size of 2 nm. The Spectralon SRS-99-010 diffuse reflectance standard served as a reference. Optical band gap energies were determined from the obtained spectra using eq 1.^33,36^ Subsequently, the DRS spectra were transformed using the Kubelka–Munk function, as defined by eq 2(36,37)
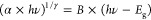
1where, α is the absorption coefficient, *h* is the Planck constant, ν is the frequency of the photon, γ is a constant, which takes a value of 2 for indirect and 1/2 for direct transition, *B* is a constant, *E*_g_ is the band gap energy.
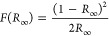
2where, *R*_∞_ is reflectance, *F*(*R*_∞_) is the Kubelka–Munk function.

Photoluminescence spectra were measured at room temperature by means of a spectrofluorometer Hitachi F7000 equipped with a xenon lamp (excitation source) and an R928 photomultiplier detector. The photoluminescence was excited at 340, 380, 400 nm (exc/em slit width of 5/5 nm) and corrected according to photomultiplier sensitivity. The SCHOTT GG455, GG400, GG365 long pass filters were applied to filter out excitation light scattering.

Raman spectra were collected using a Raman microscope, WITec Alpha 300, equipped with an air-cooled solid-state laser operating at 532 nm, a 600 grooves per nm grating, and a charge coupled device (CCD) detector. The microscope was coupled with a laser and a spectrograph via a single-mode optical fiber with a diameter of 50 μm. Samples were subjected to illumination with an output laser power of 5 mW through a 40× air objective (numerical aperture (NA): 0.6). A total of 100 accumulations were gathered, with an integration time of 0.5 s, covering the spectral range of 0–4000 cm^–1^ and maintaining a spectral resolution of ca. 3 cm^–1^. From 5 to 10 Raman spectra were acquired from randomly selected points on the sample surface, and if the acquired spectra exhibited a similar spectral profile, they were then averaged.

Electrochemical and photoelectrochemical measurements were conducted in a three-electrode system, wherein a saturated calomel electrode (SCE), platinum foil, and Co-WO_3_ samples served as a reference, counter, and working electrodes, respectively. Semiconducting properties of the modified anodic tungsten oxide layers were investigated through Mott–Schottky analyses performed in the dark. The measurements were carried out at the constant frequency of 200, 500, and 1000 Hz in a 0.1 M KNO_3_ solution using a Gamry Instrument Reference 3000 potentiostat. Photoelectrochemical tests were carried out using a photoelectric spectrometer (Instytut Fotonowy, Poland) equipped with a 150 W xenon arc lamp in a Teflon cell with a quartz window. The photocurrent vs time curves were recorded at 1.6 V vs reversible hydrogen electrode (RHE) under solar or monochromatic light. A pulse illumination in the range of 300–600 nm with a 10 nm wavelength step and 10 s light and 10 s dark cycles was employed. Measured photocurrent density values were converted into the incident photon to current efficiency (IPCE) using the following eq 3(33,38)
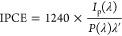
3where *I*_p_(λ) is the photocurrent density (A·m^–2^) at the wavelength λ (nm), *P*(λ) is the incident power density of light (W·m^–2^) at the wavelength λ (nm), and 1240 is a constant (W·nm·A^–1^).

Solar illumination experiments were carried out using an AM 1.5 G standard sunlight filter and a 150 W xenon light source (Instytut Fotonowy, Poland), coupled with a PalmSens4 potentiostat in a 0.1 M KNO_3_ solution. For identification of reactive oxygen species, spin-trapping experiments with 5,5-dimethyl-1-pyrroline N-oxide (DMPO) were performed. 2.5 mL of DMPO (2.5 mL 40 mg·L^–1^ solution) was added to 10 mL of the basic electrolyte, then WO_3_ and Co,F-WO_3_ were irradiated for 30 min by polarizing the electrodes at 1.6 V vs RHE. Spectra were recorded with a MiniScope MS 400 (Magnettech) at room temperature with 9.4 GHz MW frequency and sweep time of 60 s (0.05 s time constant). Samples were scanned six times to increase a signal-to-noise ratio. The electron paramagnetic resonance (EPR) parameters of the resultant spin adducts were determined by computer simulation using the EPRsim32 package.^39^ For scavenger experiments, 10 vol % of ethanol (EtOH), ethylene glycol (EG), glycerin (GY), isopropyl alcohol (IPA), and 0.01 M of sodium oxalate and sodium acetate were introduced to the solution. All chronoamperometric measurements were conducted at 1.6 V vs RHE (pH was adjusted for every used substance).

## 3 Results

**Figure 1:**
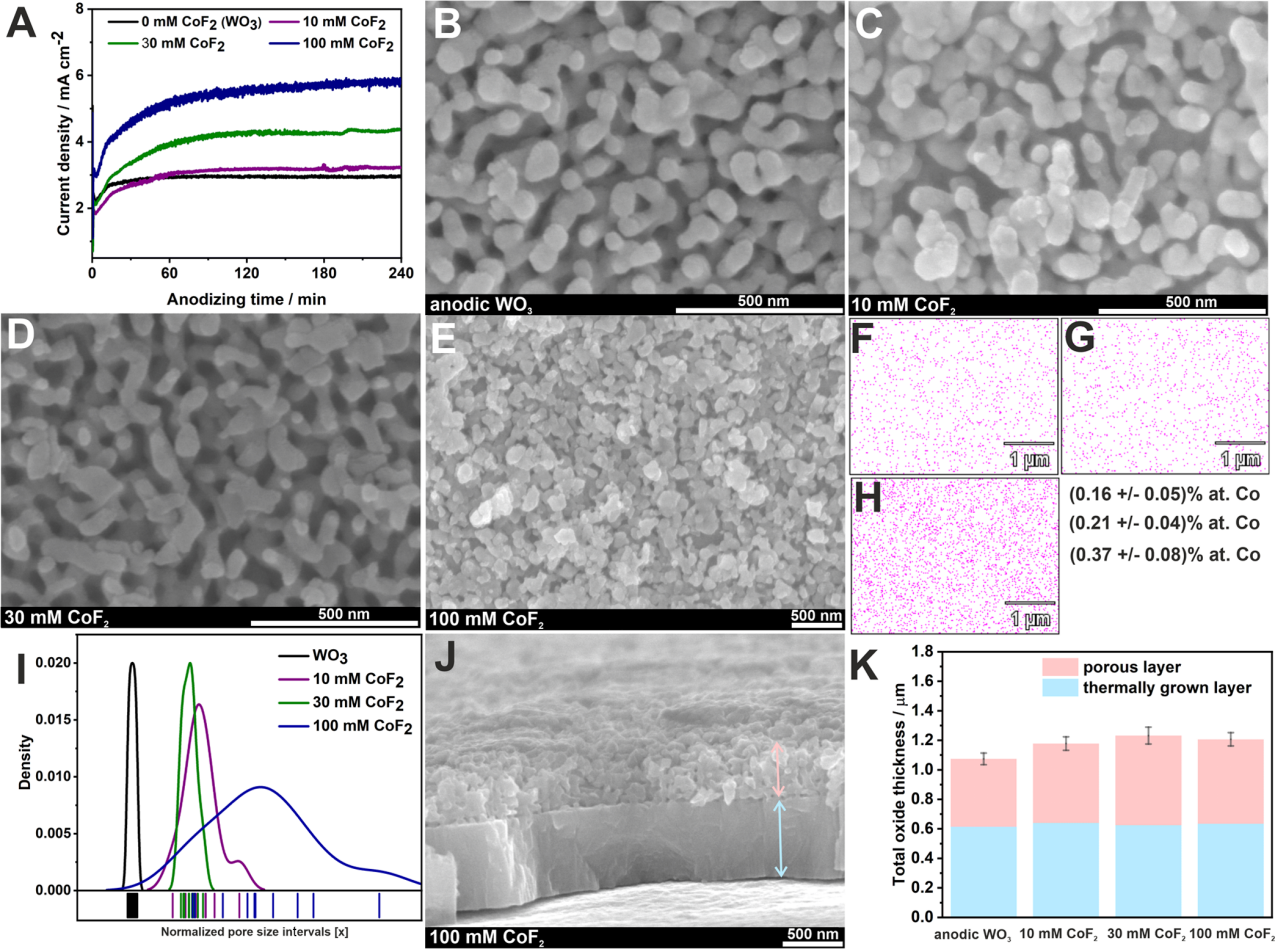
Current density vs time curves recorded during tungsten anodization in the electrolyte containing varying concentrations of CoF_2_ (A). SEM images of annealed tungsten oxide layers obtained in 1 M (NH_4_)_2_SO_4_ and 0.075 M NH_4_F at 50 V for 4 h (B), and those obtained with the addition of 10 mM CoF_2_ (C), 30 mM CoF_2_ (D), and 100 mM CoF_2_ (E). Corresponding cobalt energy dispersive spectroscopy (EDS) distribution maps for the SEM images of materials obtained with the addition of 10 mM CoF_2_ (F), 30 mM CoF_2_ (G), and 100 mM CoF_2_ (H). Distribution profile of walls sizes for all tested materials (I). The SEM cross-sectional image of the annealed Co-WO_3_ layer obtained in the electrolyte with 100 mM CoF_2_ (J). The total oxide thickness of prepared annealed materials (K).

Typical current density vs time curves recorded during anodic oxidation of tungsten without and with addition of CoF_2_ are presented in Figure 1A. Briefly, during anodic tungsten oxide formation three main stages can be highlighted. At the first few seconds, observed local minimum current values indicate the formation of the compact oxide layer over the exposed tungsten surface. The progressive thickening of the oxide layer is evidenced by the exponential decay in ionic current, followed by an increase in electronic current. This rise in electronic current results from the migration of F^–^ and OH^–^, subsequently reducing the resistance of the oxide film. The oxidation of O^2–^ or OH^–^ at the metal/oxide interface leads to the formation of oxygen bubbles, while the flow of oxide around these bubbles gives rise to the inception of pore embryos.^15^ At the final stage, the current density slightly decreases ultimately reaching a steady-state value where both ionic and electronic currents stabilize.^40^ It is widely recognized that fluoride ions play a crucial role in facilitating pore formation,^41^ as evident in the current density–time curve where the current density increases when reached the local minimum.^13^ This behavior is attributed to fluoride ions engaging in oxide dissolution reactions. As expected, increasing concentration of CoF_2_ in the electrolyte results in higher current densities observed during anodization, owing to the enhanced conductivity of the electrolyte.^13^ The as-received anodic layers were subjected to annealing under previously optimized conditions,^34^ and the morphology of anodic tungsten oxide layers and Co-doped WO_3_ obtained in the electrolyte with 10, 30, and 100 mM CoF_2_ is presented in Figure 1B–E, respectively.

**Figure 2:**
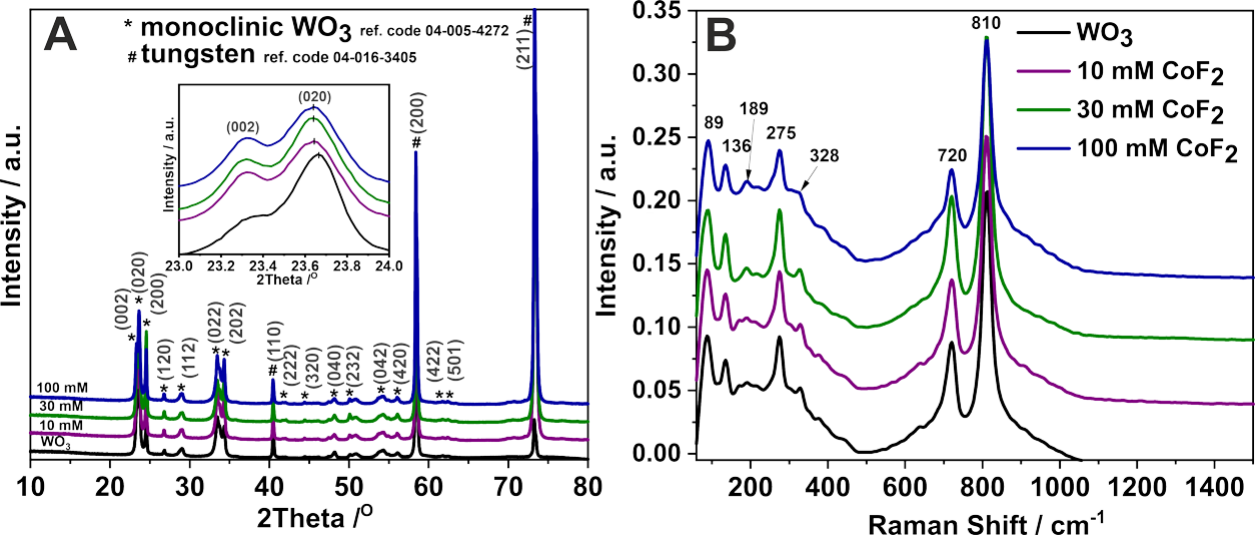
XRD patterns (A) and Raman spectra (B) of all tested materials.

Additionally, EDS mapping was performed for these samples, and the distribution of Co on the surfaces is shown in Figures 1F–H. The conducted tests suggest that a higher concentration of cobalt fluoride in the electrolyte results in an increased cobalt content in the anodic layer, ranging from about 0.1 to 0.3 atom %. The varying precursor content (0, 10, 30, and 100 mM CoF_2_) also influenced the morphology of the obtained layers, especially the average wall/ligament size which changes to about 64.8 ± 23.5, 75.5 ± 17.3, 64.1 ± 7.1, and 139.8 ± 54.6 nm, respectively. The distribution of the measured wall sizes in different fragments of materials is shown in Figure 1I. The well-defined morphology was lost for the material obtained in the electrolyte with the highest precursor concentration. This is likely attributed to the increased chemical dissolution rate of the oxide films in the anodizing electrolyte containing a high concentration of fluoride ions, leading to supersaturation conditions and precipitation of hydrated tungsten oxide on the surface of the anodic film.^13^ The precipitate subsequently sintered as a result of annealing. Moreover, SEM cross-sectional images of annealed Co-WO_3_ layers (obtained in the electrolyte with 100 mM CoF_2_, Figure 1J) reveal the previously observed thermally grown oxide layer (marked in blue) underneath the annealed porous layer (marked in pink), as reported in previous studies.^15,33,34^ The overall oxide thickness (Figure 1K) of the prepared annealed materials does not exhibit significant variation with a precursor concentration (∼1.2 μm) and is slightly higher than that of unmodified WO_3_ material (∼1.1 μm). Further characterization of the anodic layers was carried out using XRD (Figure 2A) and Raman spectroscopy (Figure 2B).

The XRD patterns exhibit two high-intensity peaks corresponding to the (200) and (211) planes of the metallic tungsten substrate (ref code 04-016-3405). Additionally, diffraction peaks indexed as (002), (020), (200), (120), (112), (022), (202), (320), (040), (232), (042), (420), (422), and (501) can be assigned to the monoclinic tungsten oxide phase (ref code 04-005-4272). Moreover, the (110) and (101) tetragonal planes of CoF_2_ (PDF 33-0417) are typically observed at angles of 26.83 and 34.14°, corresponding to the (110) and (101) peaks, respectively.^42^ These peaks may overlap with those observed for anodic WO_3_ layers.^41^ To address this, a ratio between the (120) and (202) WO_3_ planes (which may overlap with (110) and (101) planes of CoF_2_) was calculated. The obtained values of 0.27, 0.45, 0.57, and 0.64 for anodic WO_3_ layers and samples obtained with the addition of 10, 30, and 100 mM CoF_2_ to the electrolyte, respectively, suggest the possible presence of CoF_2_ in the studied materials. Furthermore, a characteristic shift of the reflection maxima (Figure 2, inset) toward lower angles was detected suggesting a change in the material’s crystal structure possibly caused by difference in the size of the host lattice (66 pm) and the dopant (74.5 pm).^43^ The composition of materials was also analyzed using Raman spectroscopy. The collected spectra show six characteristic peaks for monoclinic WO_3_ (810, 720, 328, 275, 136, and 89 cm^–1^)^44−46^ and one low-intensity peak (189 cm^–1^) for each sample. The peaks at high and medium frequencies are assigned to stretching and deformation modes, while those below 200 cm^–1^ are attributed to lattice modes.^44−46^ The peaks at 810 and 720 cm^–1^ are associated with O–W–O stretching vibration modes, while those at 328 and 275 cm^–1^ are induced by O–W–O bending modes. A low-intensity peak at 189 cm^–1^ could correspond to a characteristic peak of Co_3_O_4_ spinel, which might appear due to possible oxidation during the Raman measurement.^47^ However, the absence of other characteristic peaks such as those at 479, 515, and 617 cm^–1^, or a more intense one than 189 cm^–1^ at 686 cm^–1^,^47,48^ suggests that this is more likely related to the lattice mode of tungsten oxide, similar to the peaks at 136 and 86 cm^–1^.

**Figure 3:**
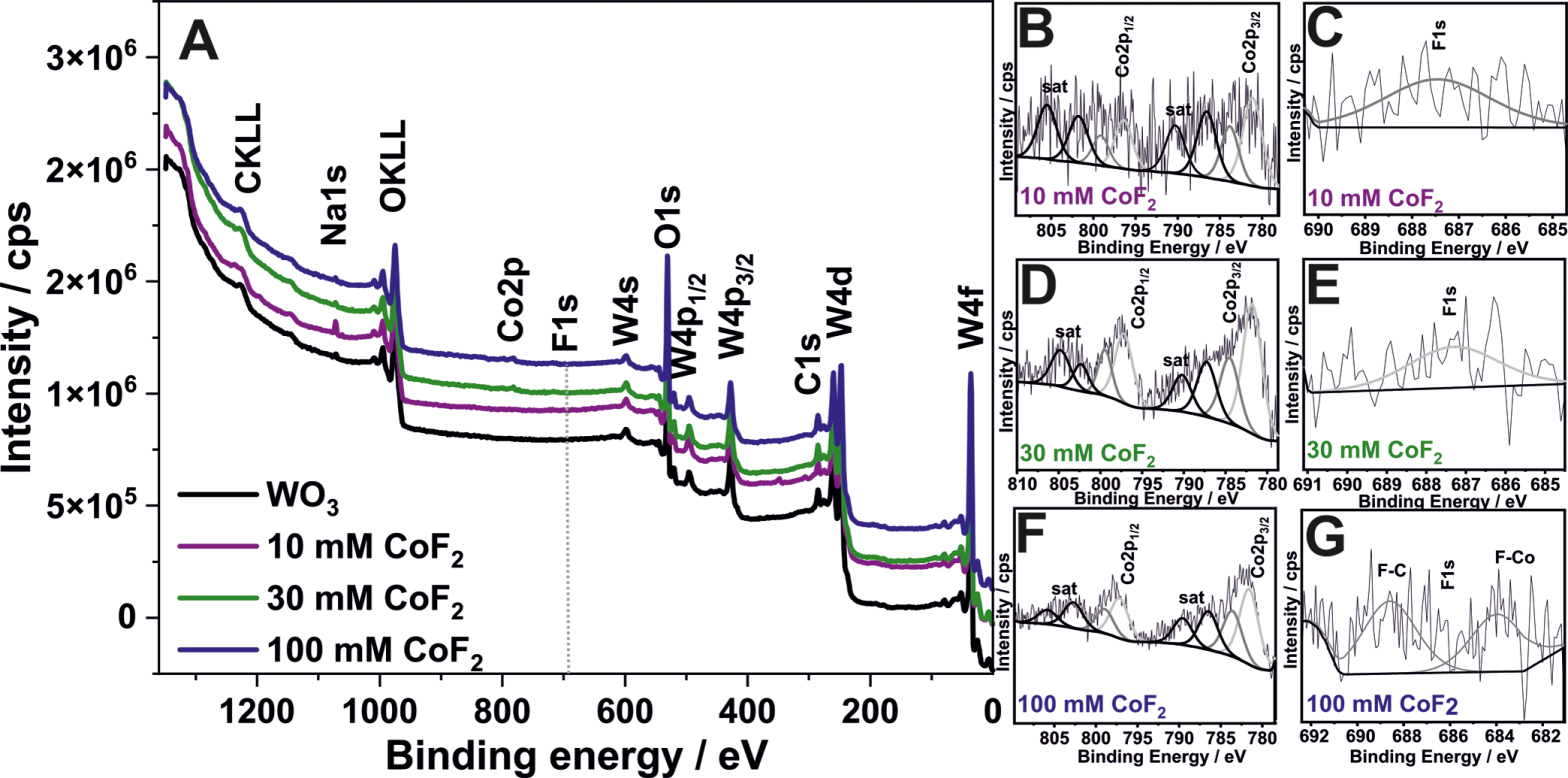
XPS survey spectra (A) and high-resolution spectra of Co 2p (B, D, F) and F 1s (C, E, G) for anodic WO_3_ layers in situ doped with CoF_2_ and annealed in air at 500 °C for 2 h.

**Figure 4:**
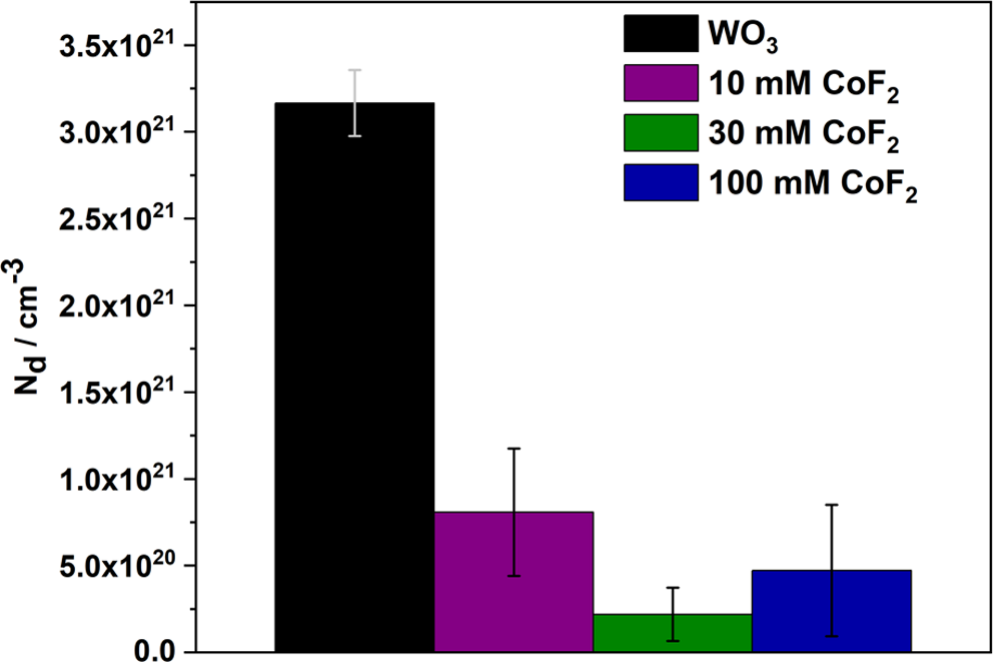
Average donor density estimated based on the Mott–Schottky relation for all studied samples.

The XPS investigations into the chemical composition of the surface of produced materials confirmed the presence of W, O, Co, C, as well as trace amounts of Na and F, as demonstrated in the survey spectra (Figure 3A). A detailed examination of the high-resolution spectra indicated that the predominant component of the oxide layers is WO_3_. The characteristic doublet for the W 4f peak was observed at a binding energy of 35.0 eV (±0.1 eV) for the spin–orbital 4f_7/2_ and 37.1 eV (±0.1 eV) for the spin–orbital 4f_5/2_ (Supporting Information, Section 1).^49^ Furthermore, it was observed that the chemical state of Co and F remained relatively unchanged despite varying concentrations of CoF_2_ in the electrolyte. For the Co 2p peak (Figure 3B,D,F), across different samples, the primary signal after deconvolution, at energies of 781.2 eV (10 mM CoF_2_), 781.8 eV (30 mM CoF_2_), and 781.6 eV (100 mM CoF_2_), could be attributed to the Co^2+^ state.^50,51^ Another notable peak at higher binding energies, above 783.0 eV, is related to the presence of Co–F bonds, identifiable as cobalt fluoride (CoF_2_).^52^ This interpretation is further supported by the main fluorine signal F 1s (Figure 3C,E,G) which displayed a maximum binding energy ranging from about 684.0 eV (100 mM) to about 687.5 eV (10 mM), confirming the presence of cobalt fluoride in the materials.^52−55^ Similar to the findings of the EDS study (Figure 1F,G,H), an increase in the Co concentration was observed in correlation with the variation in the amount of CoF_2_ added to the electrolyte. The Co content in the oxide layer ranged from 0.2 atom % (10 mM) to 0.3 atom % (100 mM). The cobalt doping was further confirmed through electrochemical investigation utilizing Mott–Schottky analysis (for details see Supporting Information, Section 2). Generally, the literature on tungsten oxide-based materials synthesized by various methods reports the donor density (*N*_d_) in the range of 10^19^–10^22^ cm^–3^.^13,15,56−58^ In this study, the initial donor density for anodic tungsten oxide was estimated at 3.2 × 10^21^ cm^–3^. All tested Co-WO_3_ layers exhibited lower *N*_d_ values (below 1 × 10^20^ cm^–3^), indicating successful substitution of W^6+^ sites in WO_3_ with cations with lower valence (Co^2+^/Co^3+^), while oxygen vacancies are formed without generating W^5+^ sites (Figure 4).^15,43,59,60^ Moreover, a linear dependence of capacitance of the space charge region versus applied potential (Figure S2) was characterized by the presence of a positive slope of the curve, indicating the n-type semiconducting behavior of all studied materials. No clear difference in the flat band potential was observed, and slight changes (∼20 mV) are likely associated with the nanostructured nature of the films.^61^

**Figure 5:**
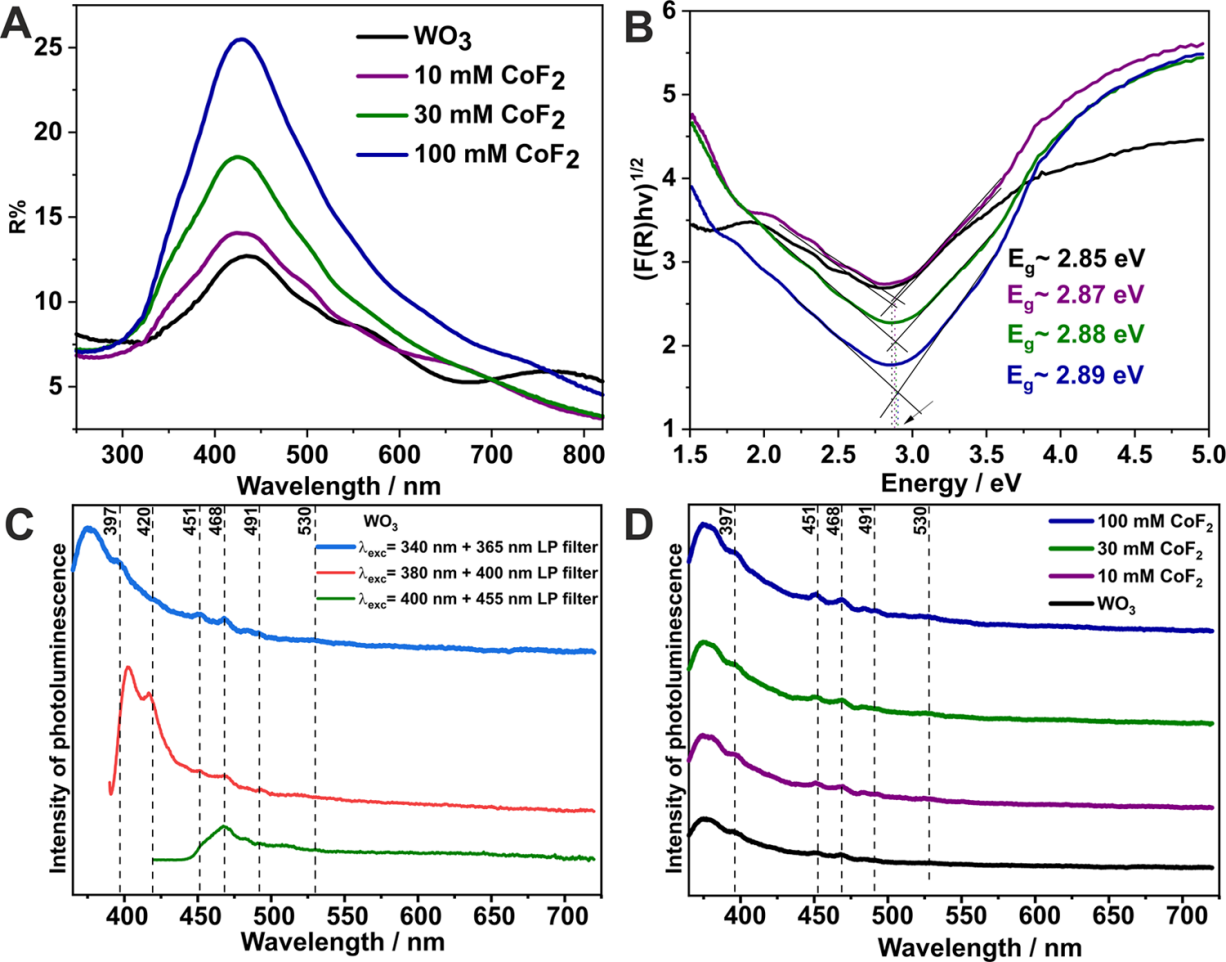
UV–vis DRS spectra (A) and corresponding Tauc’s plots (B) for all tested materials. Photoluminescence spectra of WO_3_ measured under three independent excitation wavelengths: 340, 380, and 400 nm. The LP filter—long pass filter (C). Effect of CoF_2_ on photoluminescence spectra recorded for anodic WO_3_-based materials (D).

In the next step, the optical properties of obtained materials were studied. It was observed that the Co-doped materials exhibit lower absorption compared to undoped oxide, although in the deep UV region (∼300 nm) and at the visible light edge (∼700 nm), the Co-WO_3_ materials show slightly higher absorption (Figure 5A). Optical band gaps were determined from Tauc’s plots, fitting two curves to linear fragments of the function and calculating the value from their intersection (Figure 5B).^36^ The obtained band gaps energies, while not significantly different from each other, exhibit a slight shift toward higher energies, likely attributed to a low dopant content (∼2.9 eV). Hariharan et al.^62^ reported that cobalt doping of WO_3_ powders widens the band gap from 2.81 to 3.19 eV for WO_3_ and 5 wt % Co-doped WO_3_ for unannealed materials, respectively. This observation is associated with the Burstein–Moss effect,^62^ wherein the bang gap energy increases when all states close to the conduction band become populated, resulting in the absorption edge shift. This phenomenon is observed for a degenerate electron distribution found in some degenerate semiconductors, which behave more like metals than semiconductors due to a high level of doping.^63^ Conversely, Mehmood et al.^64^ demonstrated that band gap narrowing during Co-doping might also occur (from 2.55 to 2.49 eV after introducing 8% Co) for nanoplates materials.

Photoluminescence spectra of the starting anodic WO_3_ material (without CoF_2_), measured under three independent excitation wavelengths of 340, 380, 400 nm, are presented in Figure 5C. Studying the spectrum under 340 nm light excitation, several peaks are observed that also appear at other excitation wavelengths, confirming the presence of each transition. Specifically, the blue spectrum in Figure 5C consists of bands with emission maxima located at 397, 420, 451, 468, and 491 nm with a barely visible peak at 530 nm. These wavelengths correspond to the energy transitions between conduction and valence bands or transitions between defect levels.

**Figure 6:**
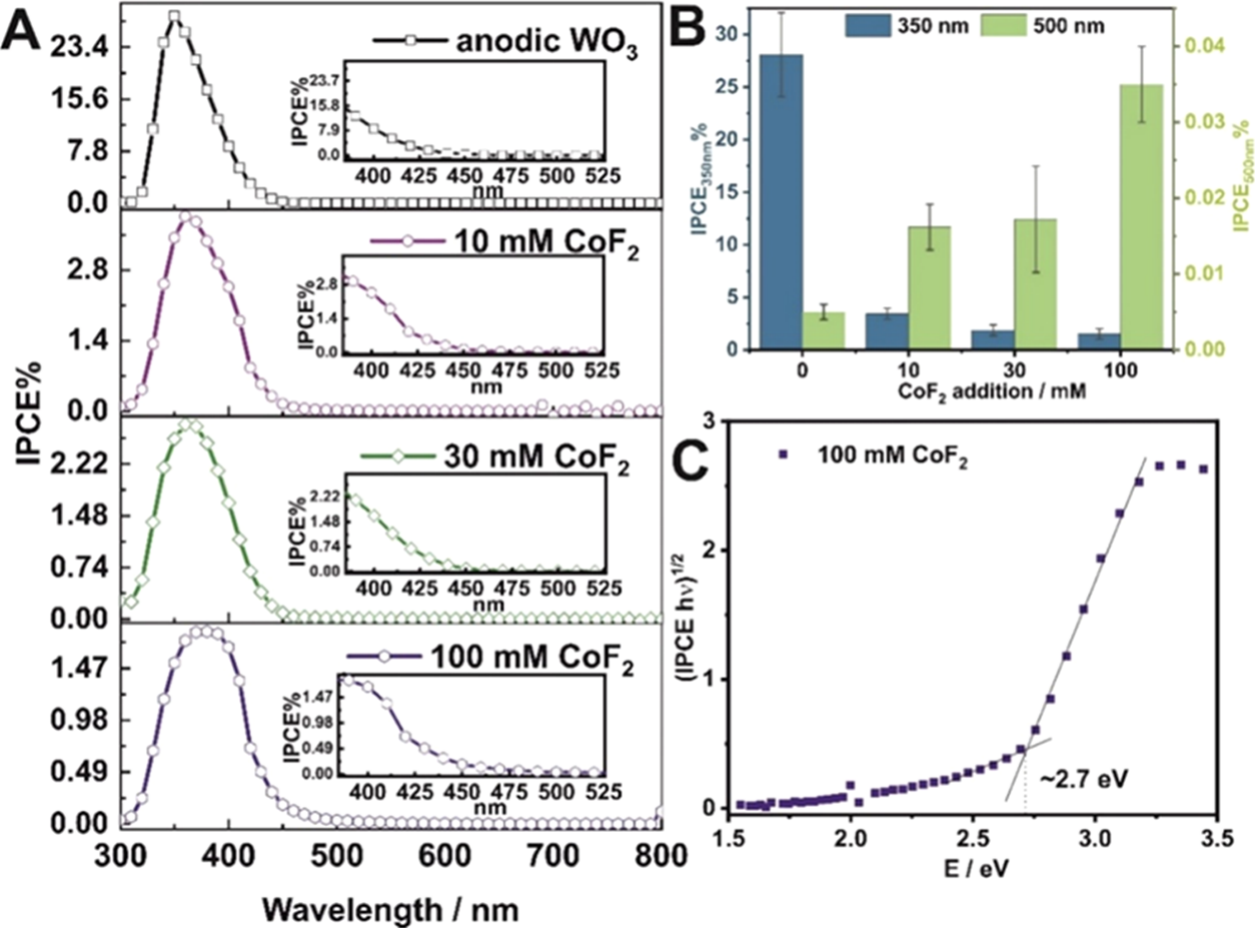
IPCE vs wavelength spectra calculated for all studied materials (A). IPCE values at 350 and 500 nm as a function of CoF_2_ concentration in the electrolyte (B). Estimation of electrochemical band gap from IPCE measurements for the sample anodized in the presence of 100 mM CoF_2_ (C).

Different defect levels arise in the electronic structure of WO_3–*x*_ as a result of the formation of V_o_^0^, V_o_^1+^, V_o_^2+^ defect states brought about by the creation of oxygen vacancies in WO_3–*x*_.^65^ Particularly, V_o_^2+^ oxygen vacancy is responsible for the formation of a resonant defect state in the conduction band, located slightly above the bottom of this band.^66^ The recombination between the electron occupying the resonant defect state in the conduction band and the hole in the valence band accounts for the ultraviolet (UV) emission at 397 nm.^67^ Next, the emission at 420 nm can be attributed to the optical band gap and coincides nicely with the value obtained from UV–vis reflectance spectroscopy (2.85 eV). The remaining emission bands, in a range of 2.00–2.50 eV, may be related to various transitions between oxygen vacancy defect states. The band centered at 491 nm may correspond to (V_o_^0^)* → V_o_^1+^ transition, while the band at 530 nm can result from electron recombination between the V_o_^0^ state and (V_o_^1+^)* unrelaxed oxygen defect state.^68^ The interpretation of peaks at 451 and 468 nm is more uncertain. Both exhibit energy greater than the estimated optical transitions between V_o_ levels but slightly lower than band-to-band recombination. Due to the small energy difference between those two bands, precisely attributing them to specific transitions is challenging. Nevertheless, some scientists believe that these bands may be related to the transition between (V_o_^1+^)* → V_o_^2+^ or W^5+^ → valence band.^69^ It was also shown that the band centered at 451 nm may even be considered a band-to-band recombination.^70^Figure 5D presents the effect of CoF_2_ concentration on the photoluminescence properties of anodic WO_3_ material. As shown, the spectra do not significantly differ from each other in the presence of CoF_2_, except for the intensity of the peak at 530 nm, which slightly increases with higher CoF_2_ concentrations. This observation inspired us to perform spectral deconvolution of the spectra measured under 400 nm excitation light. The recorded spectra, along with the deconvoluted ones, can be found in Supporting Information (Figures S3–S7). A deeper insight into the deconvoluted spectra revealed that in each case, one extra band at 623 nm can be noticed. This band is commonly observed in WO_3_-based materials^69^ and reflects the existence of the V_o_^1+^ → (V_o_^2+^)* transition. It is noteworthy that the maximum of the band shifts hipsochromically (by about 10 nm), while the intensity of the band at 499 nm increases with rising amounts of CoF_2_. This behavior ultimately enhances the intensity of the band at 530 nm (see the green spectrum in Figures 5D and S1). The photoelectrochemical properties of the tested anodic WO_3_-based layers under monochromatic light at the applied polarization potential of 1.6 V vs RHE are presented in Figure 6. Surprisingly, the IPCE vs wavelength spectra revealed that the observed changes in the materials absorption properties do not directly correspond to their photoresponse under low-intensity (∼100 μW·cm^–2^) monochromatic light. Cobalt-doped samples exhibit IPCE values ten times lower than undoped WO_3_ layers in the UV region. However, the photoresponse edge is shifted to the visible light region, as illustrated in Figure 6B. A similar trend was observed in our previous studies on anodic tungsten oxide layers sensitized with CuWO_4_^71^ and Fe_2_O_3_.^72^ In both cases, the enhancement in photoelectrochemical performance in the visible light range was attributed to heterojunction formation, altering the absorption characteristics of the materials, as evidenced by band gap narrowing. In this study, the observed relation is likely due to electron trapping in metal acting as an “electron sink”.^73^ This mechanism simultaneously extends the electron lifetime,^6,74^ leading to efficient photocurrent generation very close to the absorption edge of the materials. The described effect is further corroborated by the electrochemical band gap values determined from the IPCE spectra. Figure 6C illustrates an example of the (IPCE *h*ν)^0.5^ vs *h*ν plot, where the band gap energy was estimated from the intersection of two linear parts of the curve to the energy axis. These values are lower than those determined from reflective measurements and are equal to 2.75 eV for unmodified WO_3_ and ∼2.70 eV for all Co-modified materials.

**Figure 7:**
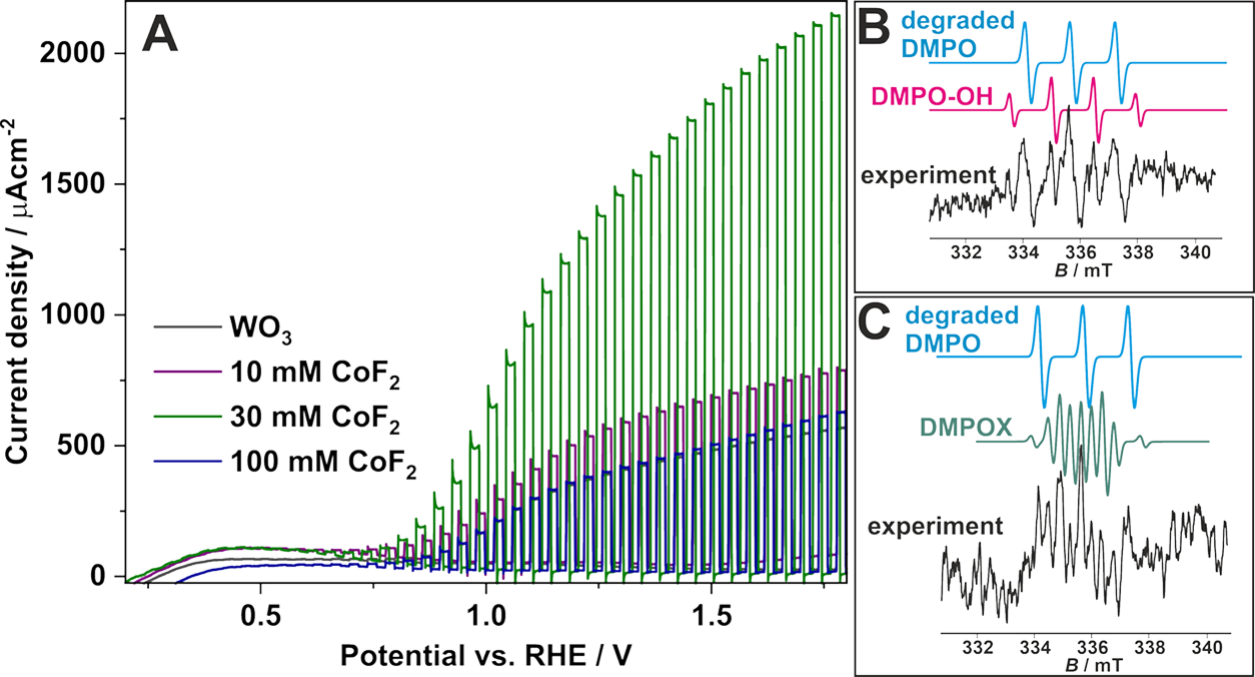
Linear voltammograms recorded in 0.1 M KNO_3_ during sequential irradiation with simulated sunlight for all studied materials (A). Experimental and simulated EPR spectra recorded after the PEC water-splitting reaction using WO_3_ (B) and Co,F-WO_3_ (C) photoanodes.

For further assessment, PEC tests under solar-simulated light of higher intensity (∼15 W·m^–2^) were conducted. Linear voltammograms recorded in 0.1 M KNO_3_ under sequential irradiation with simulated sunlight of anodic layers are presented in Figure 7. Notably, all Co-modified materials exhibited enhanced PEC activity compared to the unmodified WO_3_. Specifically, at 1.6 V vs RHE, the photocurrent densities were 510 μA·cm^–2^ for undoped WO_3_, and significantly higher for Co-doped samples: 717 μA·cm^–2^ for 10 mM CoF_2_, 1980 μA·cm^–2^ for 30 mM CoF_2_, and 550 μA·cm^–2^ for 100 mM CoF_2_. An essential aspect to consider, regarding the improved PEC performance of the Co-doped layers, is the potential catalytic role of CoF_2_,^75^ CoOOH/Co_3_O_4_^76^ in the OER.^77^ For instance, Liu et al.^75^ reported that cobalt fluoride nanorods, benefiting from a quasi-single-crystalline structure, exhibited stable and efficient OER catalytic performance without the need for additional activation procedures. In our study, a shift in the photocurrent onset potential for the modified materials (∼200 mV) was observed, moving from ∼0.16 V vs RHE for undoped WO_3_ to ∼0.39 V vs RHE for the Co-doped materials. This shift is consistent with the expected impact of the relatively low doping level on the photoelectrochemical properties of the materials. Furthermore, the impact of fluoride ions in the anodic layers on the PEC activity should be taken into consideration. In our previous studies,^34^ it was demonstrated that after heat treatment at 500 °C, no fluorine was detected in the anodic WO_3_ film. However, with the addition of CoF_2_ to the anodizing electrolyte, additional fluoride ions are present in the Co-doped materials. It has been established that fluorine-doped n-type semiconductors exhibit enhanced photocatalytic activity by promoting the generation of free ^•^OH radicals. These radicals are characterized by higher redox potential in aqueous solutions than when adsorbed on the material’s surface.^78^ Additionally, due to a strong electronegativity of F, electron-trapping properties should be considered; similarly to metal doping, the recombination rate of photogenerated electrons and holes is expected to be reduced, contributing to improved PEC performance.^78,79^ Enhanced production of ^•^OH radicals was confirmed by EPR measurements (Figure 7B,C). For the WO_3_ photoanode (Figure 7B), an isotropic signal associated with hydroxyl radicals is observed in the spectrum (a four-lines, *g* = 2.0052, *a*_N_ = *a*_Hβ_ = 1.49 mT). In the case of Co,F-WO_3_ materials used in PEC experiments, the spectrum shows seven lines that can be assigned to 5,5-dimethy-2-pyrroline-N-oxyl (DMPOX).^40^ The spin-Hamiltonian parameters of this signal, obtained by computer simulation, are *g* = 2.007, *a*_N_ = 0.73 mT, *a*_H1_ = 0.40 mT, *a*_H2_ = 0.40 mT. DMPOX is known to be formed as a product of DMPO oxidation and its presence indicates that DMPO–^•^OH adduct transforms into a more kinetically stable form. This transformation is most likely the result of the presence of a large amount of hydroxyl radicals, which causes oxidation of the spin trap. For both kind of samples, a signal consisting of three lines is observed, associated with the degradation product of the spin trap under reaction conditions.

**Figure 8:**
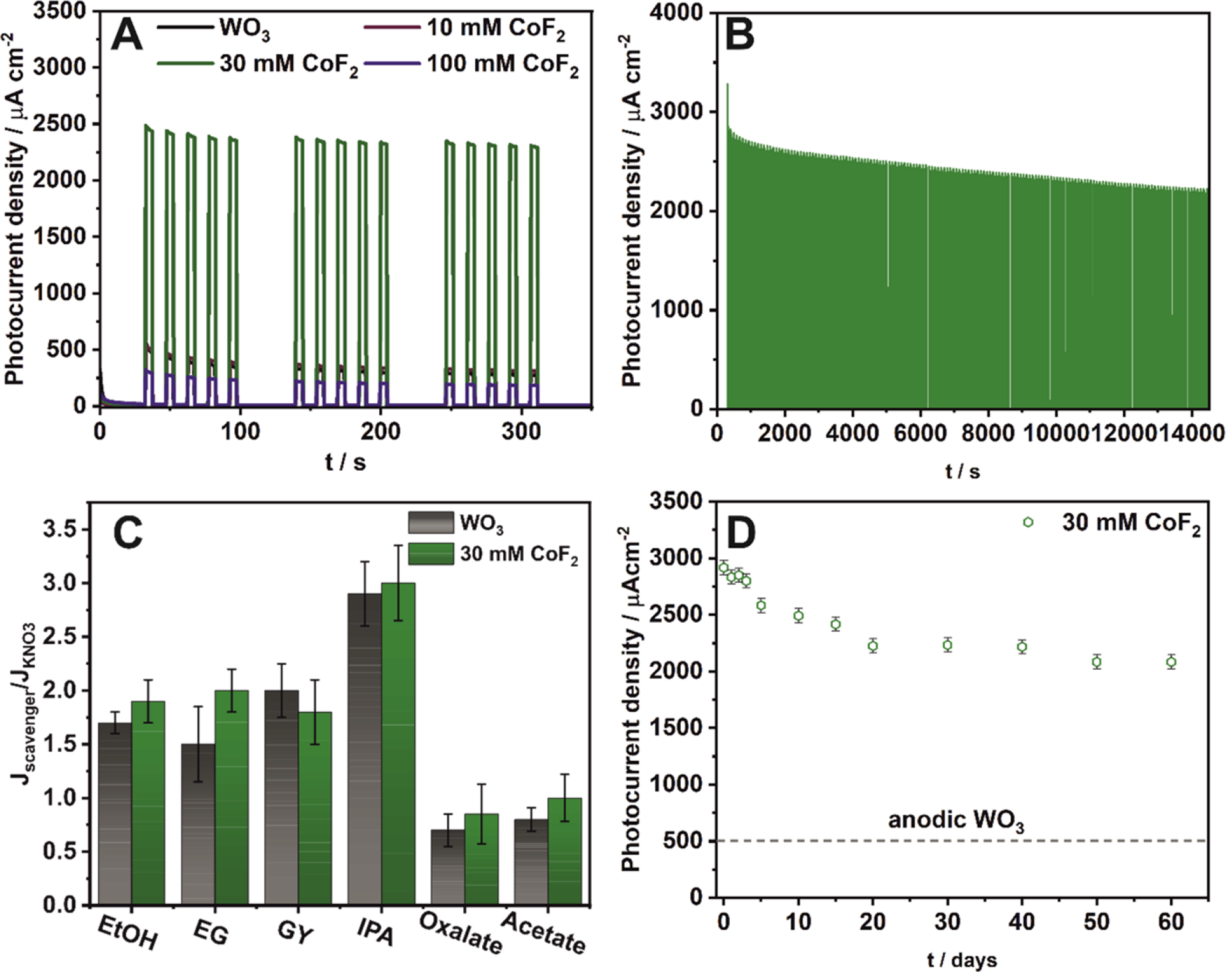
Chronoamperometric curves recorded at 1.6 V vs RHE in a 0.1 M KNO_3_ solution during sequential illumination with solar light for all tested materials (A). PEC performance of the anodic Co-WO_3_ film received via anodization in the electrolyte with the addition of 30 mM CoF_2_ during 4 h of irradiation (B). Chronoamperometric curves recorded at 1.6 V vs RHE in a 0.1 M KNO_3_ solution with the addition of a hole scavenger (ethanol) during sequential illumination with solar light for the anodic film obtained in an electrolyte containing 30 mM CoF_2_ (C). Long-term stability tests for the anodic film obtained in electrolyte containing 30 mM CoF_2_ (D).

Freshly prepared samples were assessed for their PEC performance under a constant potential of 1.6 V vs RHE (Figure 8A). Notably, the material obtained in the electrolyte with 30 mM CoF_2_ demonstrated a 5-fold higher ability to generate photocurrent, while the other samples exhibited only a marginal increase compared to undoped WO_3_. Furthermore, the stability of photocurrent over 4 h of illumination was investigated (Figure 8B). It was observed that after prolonged exposure to illumination, photocurrent values dropped by 23%; however, its activity remained significantly higher than that of undoped layers. Subsequently, the photoanode’s response to the addition of different scavengers was also tested (Figures 8C and S8). To determine the differences in the PEC mechanism, both WO_3_ and Co–F–WO_3_ were tested and, as can be seen, the improvement in PEC on individual scavengers for both electrodes is very similar, which confirms the catalytic nature of the dopant (Figure S8).

It shows also that activity of such electrode can be restored by adding a hole scavenger to the electrolyte. This minimizes recombination losses^6,80^ and contributes to the current-doubling effect.^81^ Those findings support the proposed mechanism of operation (Figure S9) of the Co–F–WO_3_ photoanode. Finally, the reproducibility of photocurrent generation in PEC water splitting was studied over 60 days of storage, revealing that the Co–F–WO_3_ anodic film can be successfully reused (Figure 8D).

## 4 Conclusions

In summary, we proposed a novel method for the single-step electrochemical synthesis of Co–F–WO_3_ anodic layers for improved photoelectrochemical water splitting. The in situ doping of anodic WO_3_ occurs during the electrochemical oxidation of metallic tungstate. The anodic layers were comprehensively characterized in terms of morphological, compositional, optical, electrochemical, and photoelectrochemical properties. It was found that wall sizes in anodic films varies with CoF_2_ concentrations. EDS analyses demonstrated a direct correlation between the cobalt content in oxide layers and the concentration of CoF_2_ in the electrolyte. XRD and Raman spectroscopy confirmed the presence of cobalt and fluorine in the studied materials, indicating the successful doping of anodic WO_3_. This was further corroborated by XPS and Mott–Schottky analysis (lower donor density values for doped samples compared to undoped WO_3_). UV–vis DRS unveiled reduced absorption in Co-doped materials, and the band gap energies demonstrated a subtle shift toward higher values (from 2.85 eV for undoped WO_3_ to 2.89 eV for the doped sample formed in the electrolyte containing 100 mM CoF_2_). Furthermore, the Co-doped samples showed lower IPCE values in the UV region but displayed a shifted photoresponse edge toward the visible light region. Photoelectrochemical tests under solar-simulated light demonstrated an enhanced PEC activity in Co-doped materials, attributed to the potential catalytic role of CoF_2_ in the OER. It was also found that the Co-doped WO_3_ layers, especially those obtained in the electrolyte with 30 mM CoF_2_, exhibited higher stability and reproducibility in long-term PEC water-splitting tests (over 40 days).

## Supporting Information Available

XPS analysis, Mott–Schottky analysis, photoluminescence results, influence of scavengers on PEC response, proposed operating mechanisms of Co–F–WO_3_ photoelectrode (PDF)
