# Supplementary material for: Surface Engineering of Anodic WO3 Layers by In Situ Doping for Light-Assisted Water Splitting
Source: ACS Appl Mater Interfaces. 2024 Jul 5;16(28):36752–62. doi: 10.1021/acsami.4c02927 (PMC11261572; doi:10.1021/acsami.4c02927)
Supplement: Supplementary file 1 — am4c02927_si_001.pdf [file am4c02927_si_001.pdf]

Supporting Information

Surface engineering of anodic WO_3 layers by *in situ* doping for light-assisted water splitting

Karolina Syrek[1]*, Sebastian Kotarba[1], Marta Zych[1], Marcin Pisarek[2], Tomasz Uchacz[1], Kamila Sobańska[1], Łukasz Pięta[1], Grzegorz Dariusz Sulka[1]

[1]*Jagiellonian University, Faculty of Chemistry, Gronostajowa 2, 30-387 Krakow, Poland*

[2]*Laboratory of Surface Analysis, Institute of Physical Chemistry, Polish Academy of Sciences, Kasprzaka 44/52, 01-224 Warsaw, Poland*

Keywords: tungsten oxide; anodization; *in situ* doping; nanostructured morphology; OER; photoelectrochemical properties

***** Corresponding author. E-mail: syrek@chemia.uj.edu.pl; karolina.syrek@uj.edu.pl

Jagiellonian University, Faculty of Chemistry, Department of Physical Chemistry & Electrochemistry

Gronostajowa 2, 30387 Krakow, Poland

XPS analysis

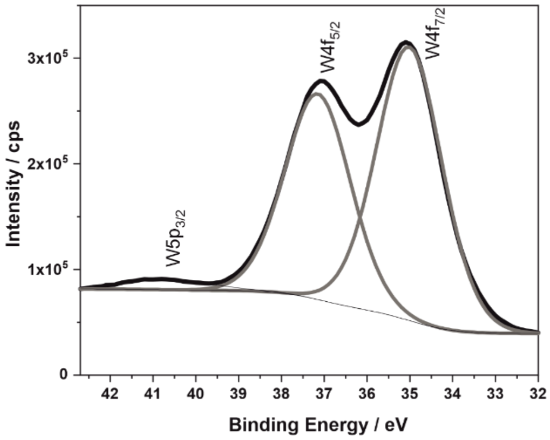

Fig. S1. The high-resolution W4f spectra of anodic WO_3.

Mott-Schottky analysis

The flat band potential and donor densities were estimated at the frequency of 200, 500, and 1000 Hz using Mott-Schottky[1] analysis (S1):

$$C_{SC}^{-2} = \left(\frac{2}{\varepsilon\varepsilon_0 q N_d}\right)\left(E - E_{fb} - \frac{kT}{q}\right) \qquad \text{(S1)}$$

where: C_{sc} is the capacitance of the space charge region ($F\cdot cm^{-2}$), N_d is the donor density (cm^{-3}), ε is the dielectric constant of porous tungsten oxide (20),[2,3] ε_0 is the permittivity of free space ($8.85\cdot10^{-14}$ $F\cdot cm^{-1}$), q is the electron charge ($1.602\cdot10^{-19}$ C), E is the applied potential (V), E_{fb} is the flat band potential (V), T is the absolute temperature (K), and k is the Boltzmann constant ($1.38\cdot10^{-23}$ $J\cdot K^{-1}$).

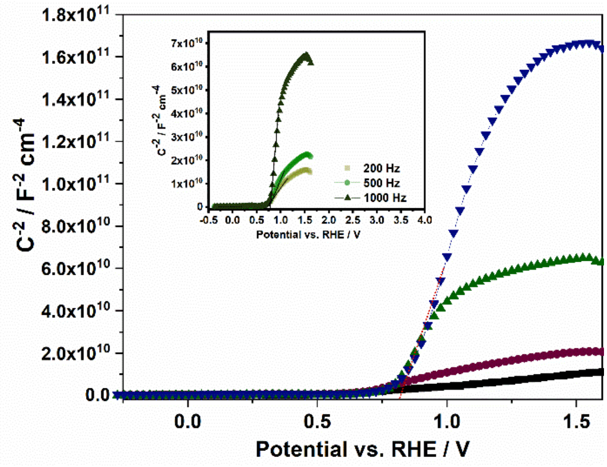

Fig. S2. Mott-Schottky plots recorded for all studied electrodes at 1000 Hz. Inset: frequency dependence for Co-F-WO_3 layers obtained in the electrolyte with the addition of 30 mM of precursor during electrosynthesis.

Photoluminescence results

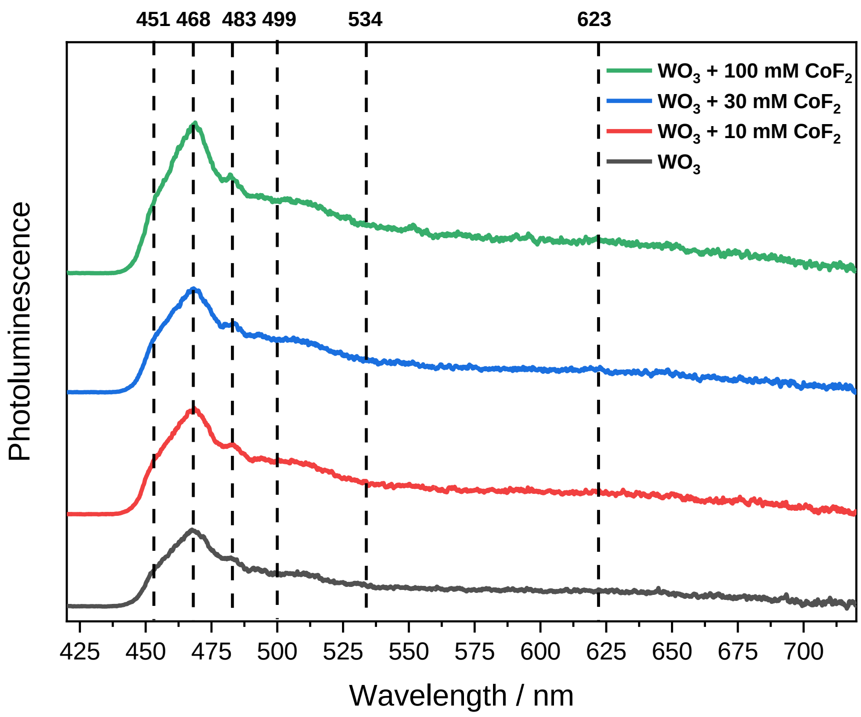

Fig S3. Photoluminescence spectra of the studied WO_3-based materials.

Peak Analysis

WO_3

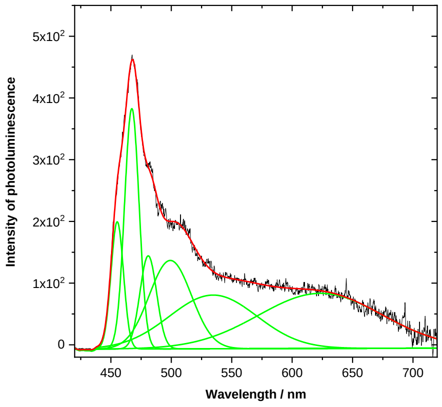

Fitting Results

Peak Index	Peak Type	Area Int	Area Intg	Center Grvt	Max Height	FWHM
1	Gaussian	2660.79	7.30087	455.24117	206.23182	12.1206
2	Gaussian	5863.17	16.0877	467.31182	389.5664	14.13901
3	Gaussian	2614.53	7.17393	480.90565	150.95925	16.27057
4	Gaussian	6274.97	17.2176	499.16414	143.35519	41.12136
5	Gaussian	7899.73	21.6757	534.57055	87.01946	85.35037
6	Gaussian	11131.7	30.544	623.33843	89.34647	120.61625

Peak Analysis

WO_3 + 10 mM CoF_2

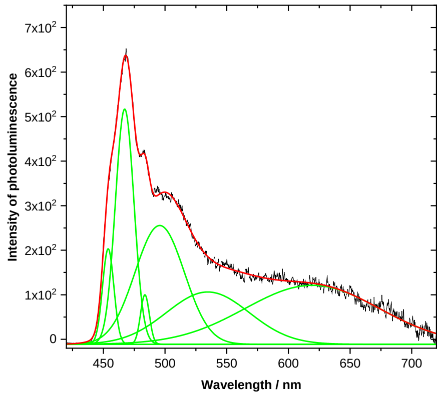

Fitting Results

Peak Index	Peak Type	Area Intg	Area IntgP	Center Grvt	Max Height	FWHM
1	Gaussian	2468.2625	4.55627	453.89132	214.37565	10.81642
2	Gaussian	9956.5882	18.37929	467.26369	528.03534	17.71395
3	Gaussian	1003.1518	1.85176	483.65353	111.05255	8.48606
4	Gaussian	13358.310	24.65867	495.65918	266.69778	47.05797
5	Gaussian	9879.9373	18.2378	534.66228	117.43728	79.05956
6	Gaussian	17506.617	32.31621	619.28868	132.45129	128.33068

Peak Analysis

WO_3 + 30 mM CoF_2

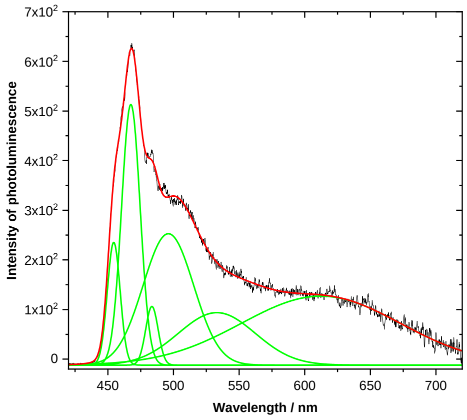

Fitting Results

Peak Index	Peak Type	Area Intg	Area IntgP	Center Grvt	Max Height	FWHM
1	Gaussian	3022.3997	5.53633	454.47049	247.30335	11.48126
2	Gaussian	9310.9197	17.05544	467.50694	524.97582	16.66177
3	Gaussian	1413.0921	2.58846	483.5468	118.12601	11.2381
4	Gaussian	12800.015	23.44665	496.09802	264.80045	45.41265
5	Gaussian	7767.2576	14.22781	532.99733	105.72446	69.02162
6	Gaussian	20278.398	37.14531	612.27485	138.95305	142.56422

Peak Analysis

WO_3 + 100 mM CoF_2

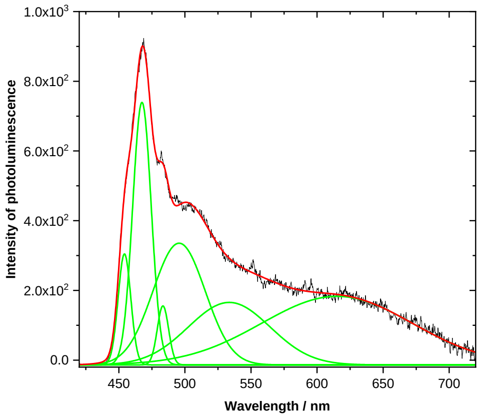

Fitting Results

Peak Index	Peak Type	Area Intg	Area IntgP	Center Grvt	Max Height	FWHM
1	Gaussian	3820.3003	4.93981	454.23101	318.27587	11.27617
2	Gaussian	13222.222	17.0969	467.4736	753.08244	16.49414
3	Gaussian	1827.7378	2.36334	483.34902	169.11913	10.15287
4	Gaussian	17335.240	22.4152	495.59195	349.13441	46.64816
5	Gaussian	13914.632	17.99221	533.51268	179.26058	72.93037
6	Gaussian	27216.843	35.19254	615.06474	196.7477	134.43878

Fig S4. Deconvolution of the spectra of the studied WO_3-based materials.

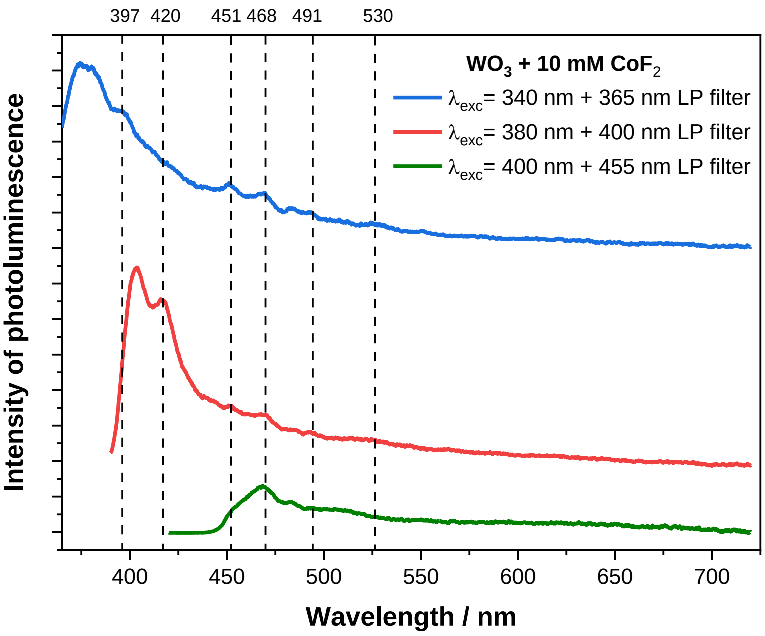

Fig. S5. Photoluminescence spectra of WO_3 + 10 mM CoF_2 material measured under three independent excitation wavelengths: 340 nm, 380 nm, and 400 nm. The LP filter – long pass filter.

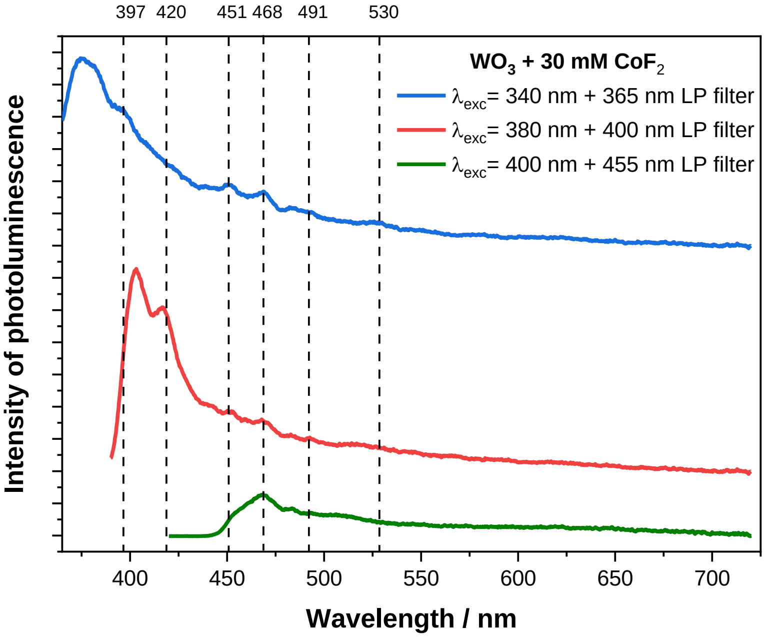

Fig. S6. Photoluminescence spectra of WO_3 + 30 mM CoF_2 material measured under three independent excitation wavelengths: 340 nm, 380 nm, and 400 nm. The LP filter – long pass filter.

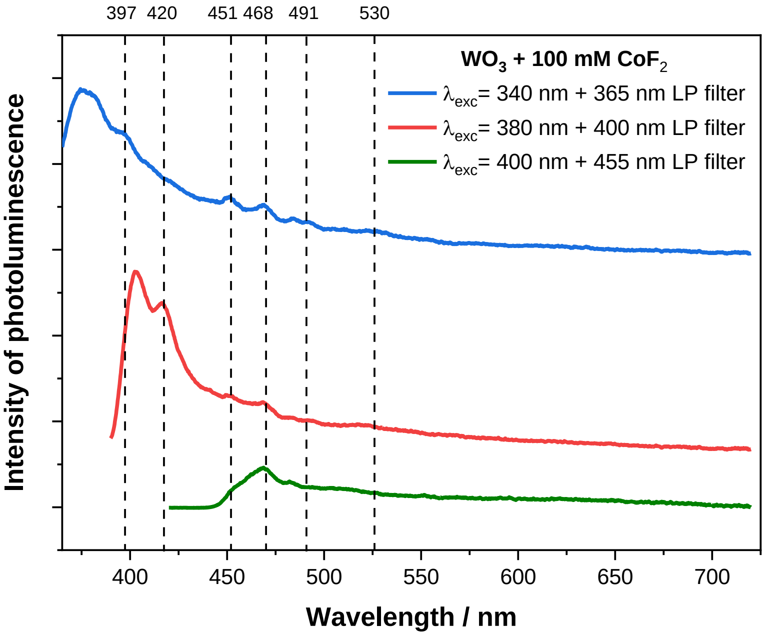

Fig. S7. Photoluminescence spectra of WO_3 + 100 mM CoF_2 material measured under three independent excitation wavelengths: 340 nm, 380 nm, and 400 nm. The LP filter – long pass filter.

Influence of scavengers on PEC response

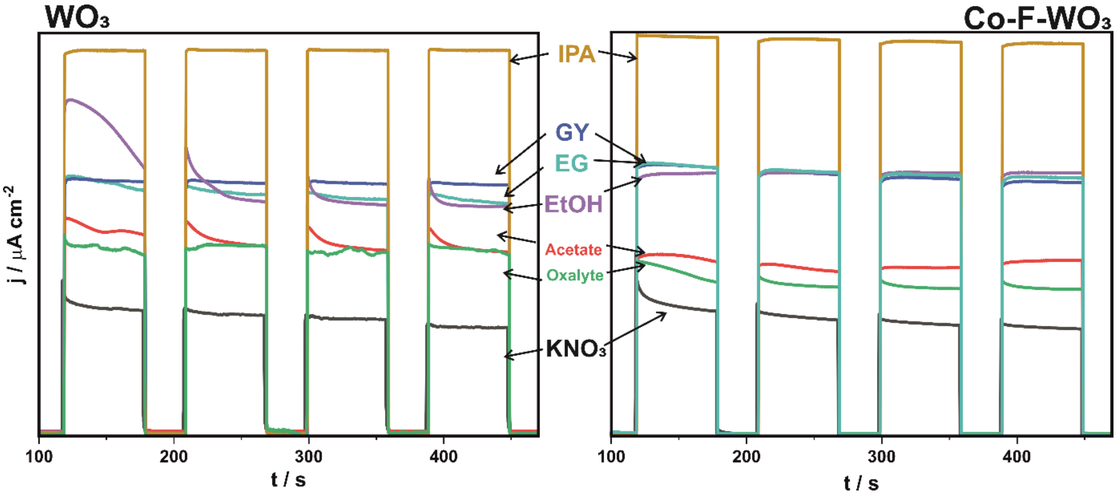

Fig. S8. PEC response of anodic WO_3 and Co-F-WO_3 photoelectrodes to different scavengers.

Proposed operating mechanisms of Co-F-WO_3 photoelectrode

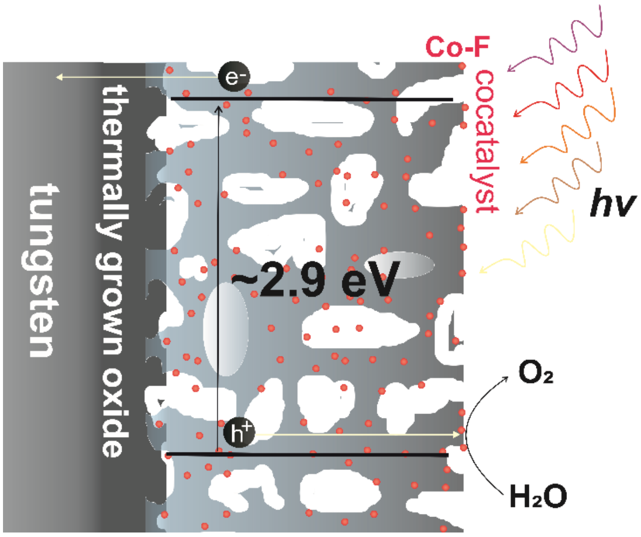

Fig. S9. Proposed PEC mechanism.

Ref.

1 K. Sivula, *ACS Energy Lett*, 2021, 6, 2549–2551.

2 J. Yan, T. Wang, G. Wu, W. Dai, N. Guan, L. Li and J. Gong, *Advanced Materials*, 2015, **27**, 1580–1586.

3 K. Syrek, M. Zych and G. D. Sulka, *Journal of Industrial and Engineering Chemistry*, 2022, **112**, 316–322.
